# Supplementary figures and images for: Calcium signals shape metabolic control of H3K27ac and H3K18la to regulate EGA
Source: bioRxiv. 2025 Mar 16:2025.03.14.643362. Preprint. [Version 1] doi: 10.1101/2025.03.14.643362 (PMC11952514; doi:10.1101/2025.03.14.643362)

Figure S1

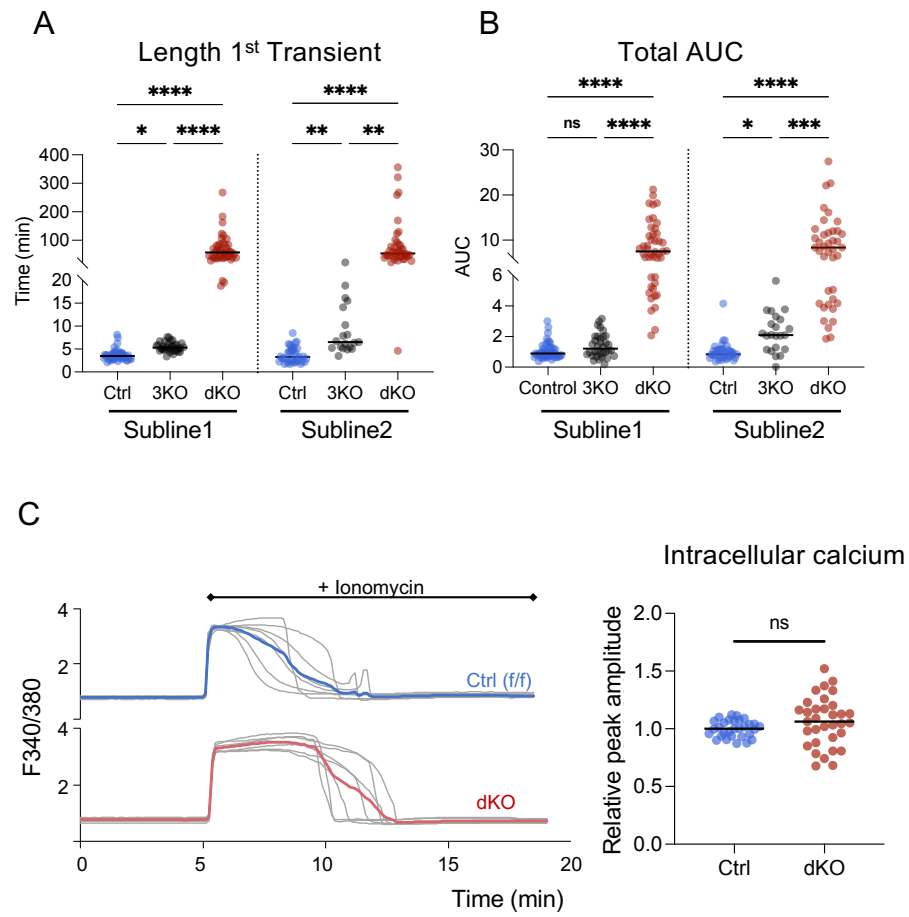

Figure S2

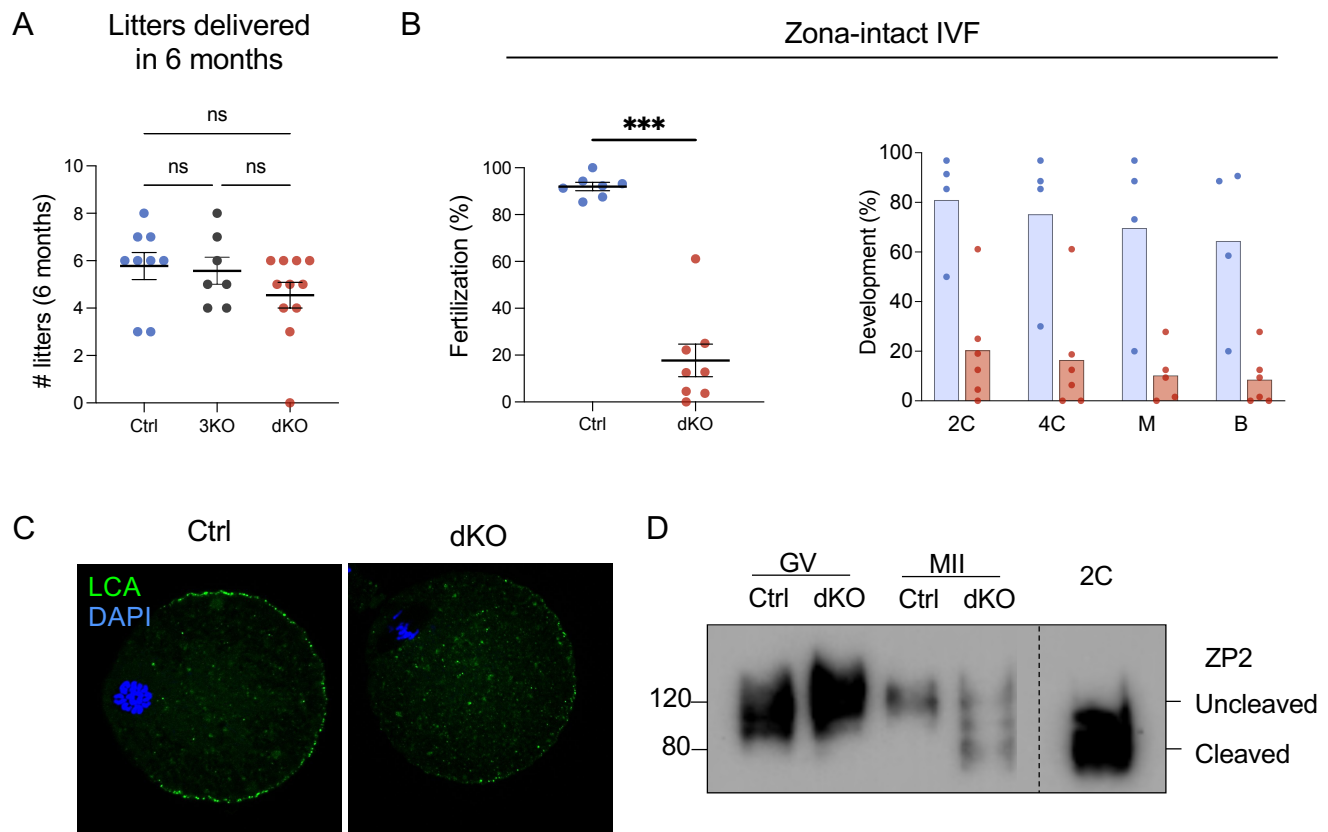

Figure S3

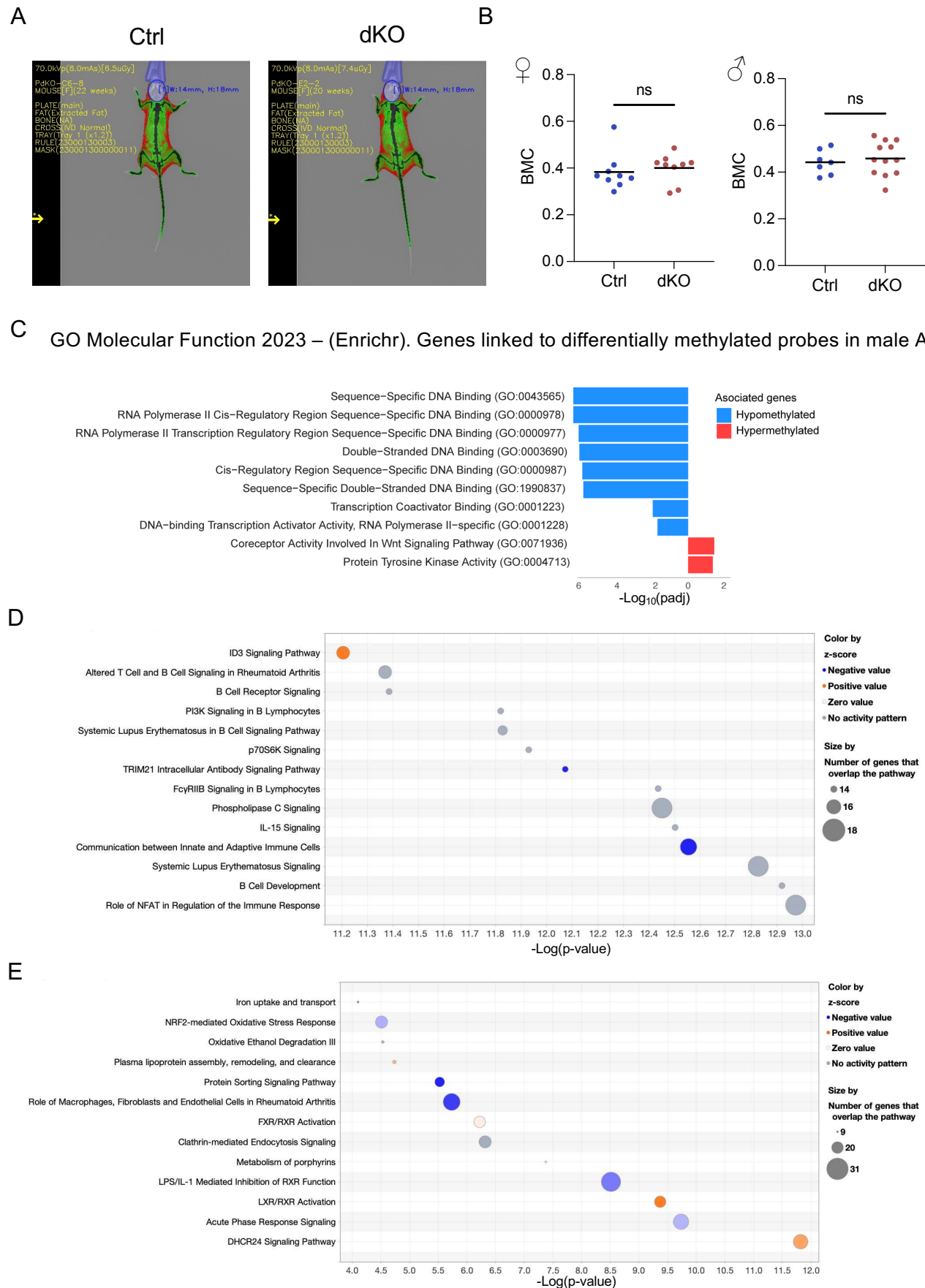

Figure S4

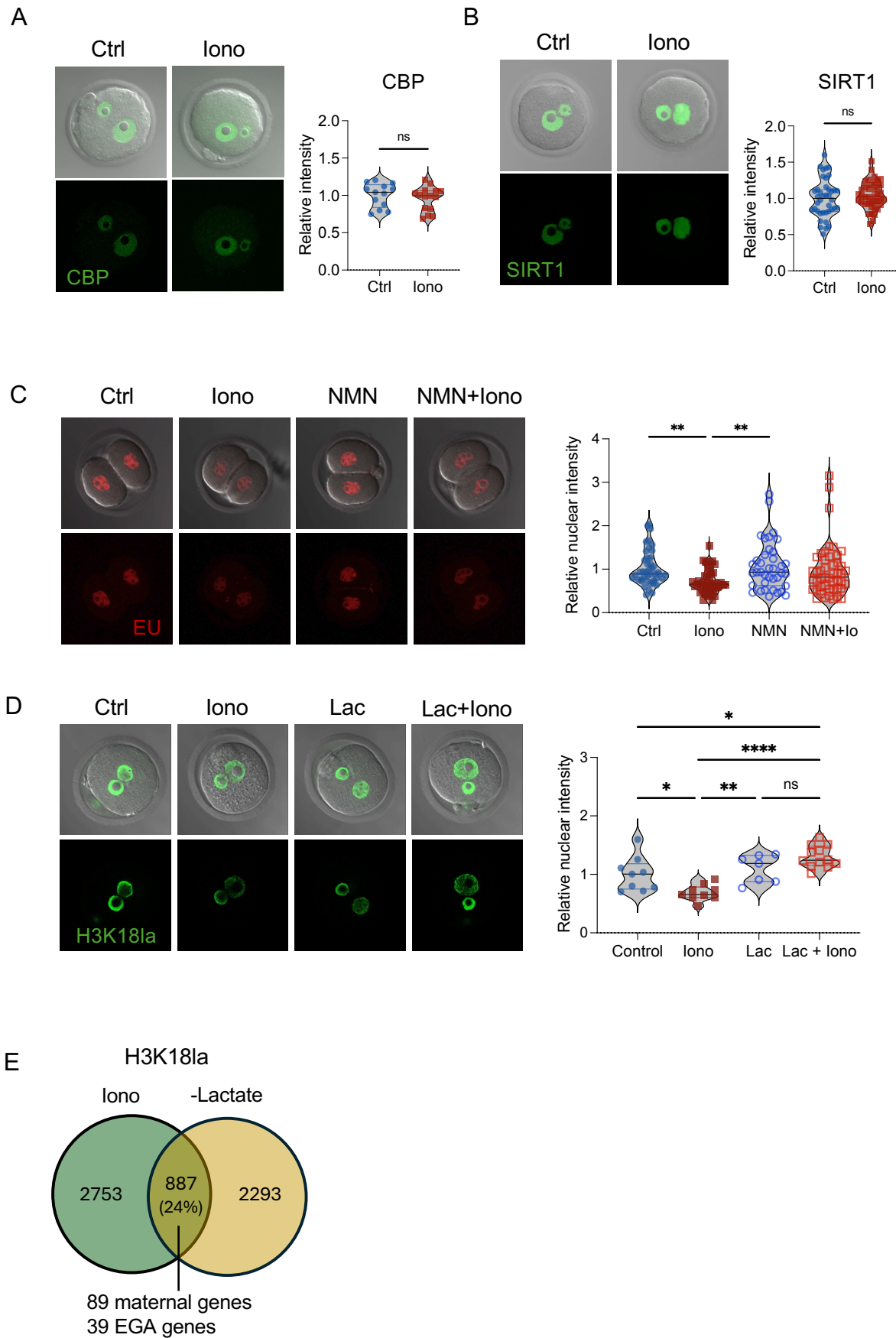

Supplement: 1 — Figure S1. Analysis of ratiometric Ca2+ imaging during IVF of control (blue), PMCA3-KO (3KO, gray) and PMCA1/PMCA3 double KO (dKO, red) eggs. N = 3 independent experiments using 2 independent mouse sublines (Subline1, Subline2). (A) Length of the first Ca2+ transient. (B) Area under the curve (AUC) of calcium signal, relative to controls. For A-C, Kruskal-Wallis with Dunnett’s multiple comparisons test was performed; *p<0.05, **p<0.005, ***p<0.0005; ****p<0.0001; ns, not significant. (C) Ionomycin-induced Ca2+ release in eggs from control (blue) and dKO (red) females. Left, representative traces; colored lines are means of the experimental traces shown in gray. Right, peak level of Ca2+ released as an indirect indicator of Ca2+ stores. T-test; ns, not significant. Figure S2. dKO eggs have premature zona pellucida hardening. (A) Number of litters born after mating Ctrl, 3KO, and dKO females to WT males; each dot represents the average litter size per female from 9, 7, and 11 breeding pairs, respectively, during a 6-month breeding trial. Kruskal-Wallis with Dunnett’s multiple comparisons test; ns, not significant. (B) Left, percentage of fertilized embryos following IVF with intact zona-pellucida. Each dot represents an independent biological replicate and horizontal bars indicate median. Mann Whitney test; ***p<0.0005. A total of 115 Ctrl and 170 dKO eggs were included in the analysis. Right, percentage of embryos that reached the various preimplantation embryo stages following IVF with intact zona-pellucida. Preimplantation embryo stages: 2C (2-cell), 4C (4-cell), M (morula) and B (blastocyst) stage embryos. (C) Representative images of LCA staining of cortical granules in zona-free MII-eggs from Ctrl and dKO females. (D) Representative immunoblot of glycoprotein ZP2 from Ctrl or dKO germinal vesicle stage oocytes (GV) or eggs (MII). Wild-type embryos at the 2C stage served as reference for ZP2 cleavage. Figure S3. Increased Ca2+ at fertilization has long term effec [file NIHPP2025.03.14.643362V1-supplement-1.pdf]
